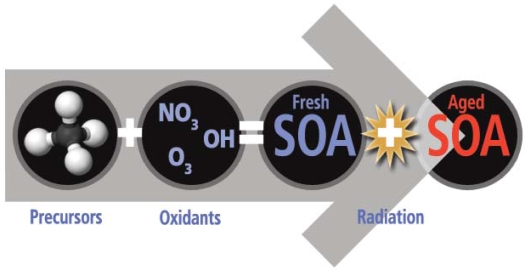# Particulate Matter: New Chapter on the Next Generation of Aerosols

**Published:** 2008-12

**Authors:** Cynthia Washam

Scientists have known for decades that carbonaceous molecules emitted by cars, factories, and ships can react chemically to form new pollutants called secondary organic aerosols (SOA). Until recently they thought these offspring constituted only a small part of the pollution plume [see “Ship Sulfate an Unexpected Heavyweight,” *EHP* 116:A475 (2008)]. So when researcher Jose-Luis Jimenez of the University of Colorado at Boulder and colleagues analyzed the particle content of the air of Riverside, California, about 50 miles east of Los Angeles, they were surprised to find that only 10–30% came from smokestacks and tailpipes—the rest was SOA generated in the atmosphere. The findings, reported 15 October 2008 in *Environmental Science & Technology*, add to growing suspicions that people may be exposed to substantial levels of unregulated health-threatening pollutants.

Organic aerosols are formed from burning coal, gasoline, and other fossil fuels. Emissions of some primary organic aerosols are regulated by the Environmental Protection Agency (EPA). SOA forms when hydrocarbons (including toluene and xylene from fuel combustion) and other lesser volatile precursors oxidize and condense onto particles in the atmosphere. SOA also forms from terpenes and sesquiterpenes emitted by vegetation. Because SOA forms more readily in sunlight, it is more prevalent during the summer and in warmer climates. SOA disperses over wide areas, often far downwind of the cities where its precursors were emitted.

Particles contribute to preterm birth, low birth weight, asthma, and cardiovascular disease, among other health effects. Researchers think SOA may also threaten human health, but they’re just starting to gather evidence to back that hypothesis. “We know a lot about what particles do, but we don’t know a lot about the role of SOA in that,” says environmental epidemiologist Joel Schwartz of the Harvard University School of Public Health. However, one clue lies in the size of SOA. “Smaller particles are more aggressive in finding their way into the lungs and crossing the blood barrier,” says atmospheric researcher Rainer Volkamer of the University of Colorado at Boulder. With a diameter of less than 1 μm, SOA is among the smaller particles in an ambient distribution.

A study in the October 2003 issue of *Inhalation Toxicology* showed a decrease in respiratory frequency in rodents exposed to ozone- and terpene-based SOA. First author Annette Rohr, senior technical manager of the Electric Power Research Institute in Palo Alto, California, says, “We had evidence of airway irritation as well as airflow limitation. There’s no reason to think humans wouldn’t have the same effect.” She notes, however, that the concentrations used in her studies were many times higher than likely human exposures.

EPA-funded studies under way in Los Angeles, Fresno, Pittsburgh, St. Louis, Boulder, Mexico City, and Chebogue Point (Canada) are looking at how emissions, temperature, wind, and other factors influence SOA formation and movement. Dan Costa, national program director for air research at the EPA, hopes that information eventually can be used to predict air quality. “We’re trying to get fundamental information we can plug into models to project the air quality in a community,” he says. “But we’re a ways away from being able to regulate these things.”

## Figures and Tables

**Figure:**